# Comparison of nostril sizes of newborn infants with outer diameter of endotracheal tubes

**DOI:** 10.1186/s12887-021-02889-5

**Published:** 2021-09-23

**Authors:** Bianca Haase, Ana-Maria Badinska, Christian A. Maiwald, Christian F. Poets, Laila Springer

**Affiliations:** Department of Neonatology, University Children’s Hospital of Tuebingen, Calwerstraße 7, 72076 Tuebingen, Germany

**Keywords:** Nasal intubation, Endotracheal tube size, Nostril size, Preterm, Newborn

## Abstract

**Background:**

Recommendations for endotracheal tube (ETT) size usually refer to the inner diameter (ID). Outer diameters (OD), however, vary greatly between manufacturers, which in some brands might cause difficulties in passing the ETT through the nostrils if choosing the nasal route for intubation. Even though the nostrils are dilatable by an ETT, it might be difficult to pass an ETT through the posterior naris (narrowest point of the nasal passage), if the OD is bigger than the nostrils. Therefore, nostril size may provide some guidance for the appropriate ETT size preventing unsuccessful intubation attempts. This study therefore compares nostril sizes of newborn infants with ODs of ETTs from several manufacturers.

**Methods:**

This is a subgroup analysis of a prospective observational study, performed in a single tertiary perinatal centre in Germany. The diameter of the nostril of infants born between 34 and 41 weeks´ gestation was measured in 3D images using 3dMDvultus software and compared to the OD of ETT from five different manufacturers.

**Results:**

Comparisons of nostril sizes with ODs of different ETTs were made for 99 infants with a mean (SD) birthweight of 3058g (559) [range: 1850-4100g]. Mean (SD) nostril size was 5.3mm (0.6). The OD of the 3.5mm ETT of different manufacturers ranged from 4.8-5.3mm and was thus larger than the nostril size of 20-46% of late preterm or term infants.

Some OD of a 3.0mm ETT were even bigger than the OD of a 3.5mm ETT (e.g. the 3.0mm ETT from Rüsch® has an OD of 5.0mm while the 3.5mm ETT from Portex® has an OD of 4.8mm).

**Conclusions:**

Clinicians should be aware of the OD of ETTs to reduce unsuccessful intubation attempts caused by ETT sizes not fitting the nasal cavity. Generated data may help to adapt recommendations in future.

**Trial registration:**

Subgroup analysis of the “Fitting of Commonly Available Face Masks for Late Preterm and Term Infants (CAFF)”-study: NCT03369028, www.ClinicalTrials.gov, December 11, 2017.

## Background

Endotracheal intubation is a common procedure in newborns [1] and can be performed orally or nasally with no difference in complication rates, e.g. malposition of the tube, accidental extubation or local trauma [1]. The nasal route is preferred over the oral intubation in many countries including Germany. An endotracheal tube (ETT) that is too big might cause complications such as subglottic stenosis [2], but might also contribute to significant time delay during nasal intubation because the ETT does not pass the nasal cavity.

An optimally sized ETT should have an inner diameter (ID) as large as possible to reduce airway resistance, since the ID increases the resistance in the fourth power [3]. The outer diameter (OD) should not be bigger than the inner diameter of the cricoid cartilage, as this has been claimed to be the narrowest part of the neonatal airway, to avoid laryngeal trauma [4]. Since the subglottic cricoid area correlates with birthweight [4, 5], weight-based recommendations are most commonly used for the correct ETT size. Alternatively, approaches using body length [6] or a formula based on gestational age (GA) have also been published [5].

The most recent European Resuscitation Council (ERC) guidelines from 2021 recommend an ETT with an ID of 3.5mm (hereinafter referred to as 3.5mm ETT) in late preterm and term infants [7–11] . Other guidelines do not provide specific recommendations on the appropriate tube size [8–11]. The statement from the International Liaison Committee on Resuscitation (ILCOR) from 1999 is imprecise [12], as it recommends a 3.0 or 3.5 mm ETT for infants weighing between 2000-3000g, and a 3.5 or 4.0mm for those weighing ≥3000g [12]. Moreover, it remains unclear on which evidence these recommendations are based.

Additionally, all recommendations refer to the ID of the ETT, although there are variations of the OD depending on the manufacturer.

The OD, however, contributes to pressure related injuries in the subglottic and nasal region [13] as well as the palatal groove [1].

Additionally, if the OD is too large, it might hinder passing the nasal region when inserting the ETT. During nasal intubation the first challenge is to pass the ETT through the nostrils and then through the posterior naris, which is the narrowest part of the nasal passage located in the narrowed connection between the nasal cavity and the nasopharynx, where choanal stenosis (CS) is usually located [14]. Although the narrowest part of the nasal passage is a few centimetres behind the nostrils, it seems unlikely to pass the posterior naris if the OD of an ETT is much larger than the nostril size [14, 15]. Therefore, nostril size may provide some guidance for the appropriate ETT size preventing unsuccessful intubation attempts.

Since nasal intubation is the preferred route for neonatal intubation in Germany, the aim of this study was to measure nostril sizes in these infants and compare them with the OD of ETTs of different manufacturers.

## Methods

### Study design and consent

This is a subgroup analysis of a previous single-centre study conducted at University Children’s Hospital Tuebingen, Germany [16]. The study was registered at www.clinicaltrials.gov (NCT03369028) and approved by the local ethics committee (approval number: 704/2017BO1). All parents gave written informed consent. The trial was performed in accordance with the Declaration of Helsinki and the guidelines of Good Clinical Practice (GCP).

### Patients

Infants born between 34 and 41 weeks´ gestation were eligible within 72 hours of birth. Exclusion criteria were dysmorphic features or the need for respiratory support. The intention was to obtain a study population that was evenly distributed among gestational age groups with at least 11 infants per week of gestation.

### Equipment and outcome

**Fig. 1:**
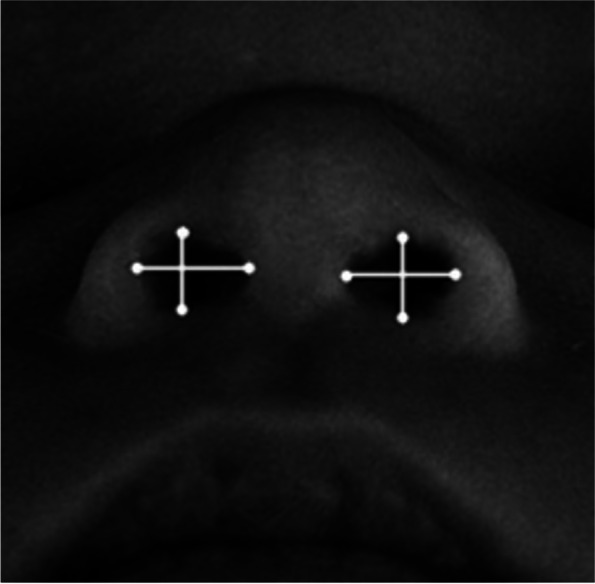
Three-dimensional image of the nose of an infant. Showing the 8 landmarks used to measure the nostril size. The nostril sizes of the left and right nostril were measured as the mean value between the two perpendicular distances for each nostril. Parental consent has been provided

3D-images were taken in a supine position and obtained using a 3dMDhead (3dMD Limited, Brentford, London, UK) system with six synchronised video cameras [16]. Length and high of the infants’ nostrils were measured using Vultus software (Software advice, Austin, Texas, USA). Eight landmarks were used: the most lateral, the most median, the most highest and lowest point of each nostril. The mean values of the two perpendicular distances for each nostril and of the right and left nostril were used for each infant (Fig. 1) and thereby mean nostril size calculated for each infant.

ODs of the ETT were collected from product information sheets from the manufacturers.

We then compared the ODs of 3.5mm ETTs with the nostril size of each infant in line with the ERC guidelines [7].

Second, in accordance with the ILCOR statement from 1999 [12], the population was divided into two groups (birthweight <3000g and ≥3000g) and the nostril size of each infant with a birthweight <3000g or ≥3000 g was compared with the OD of a 3.0 and 3.5mm and a 3.5 and 4.0mm ETT, respectively, from different manufacturers (Table 1).

**Table 1:** Tube manufacturers and its inner and outer diameter

Manufacturer	Mallinckrodt®	Vygon®	Endosid®	Portex®	Rüsch®
ID (mm)	OD (mm)	OD (mm)	OD (mm)	OD (mm)	OD (mm)
**3**	4.4	4.6	4.0	4.2	5.0
**3.5**	4.9	5.2	4.9	4.8	5.3
**4**	5.6	5.7	5.5	5.5	6.0

Mallinckrodt® contour (Athlone, Ireland), VYGON® (Ecouen, France), Endosid® (ASID BONDZ, Herrenberg, Deutschland), Portex® Blue line (smith medical, Hythe, UK), Rüsch® Safety clear (Kernen, Germany). The listing is incomplete and only serves as an orienting comparison of manufacturers. ID = inner diameter, OD = outer diameter

In each of these comparisons the ETT was considered too large for the respective infant if the OD of the ETT was larger than his/her mean nostril size.

### Statistical analysis

Continuous data are shown as mean [standard deviation (SD)] if Gaussian distributed or median [interquartile range (IQR)] if skewed. Categorical data are summarized as counts and percentages. If data followed a Gaussian distribution, Pearson’s correlation coefficient was used. P-values <0.05 were considered statistically significant. All statistical analyses were performed using SPSS (version 25, SPSS Inc, Chicago, Ill).

## Results

In total, in 100 of 102 infants of the original study 3D images were taken and in 99 infants the nostril size of at least one nostril could be analysed. One infant was excluded due to poor image quality. No infant had been intubated before the 3D image was taken. Birth weight ranged from 1850g to 4100g. Demographic and clinical characteristics are shown in Table 2.

**Table 2:** Population demographics (n=99)

Characteristics	all (n=99)
Birth weight (g)	3057.5 (558.8)^a^
Gestational age (weeks)	38.1 (2.2)^a^
Length (cm)	49 (47-51)^b^
Head circumference (cm)	34.5 (33.0-35.5)^b^
Postnatal age during study (hours)	45.8 (13.6)^a^
Male (%)	53 (54%)^c^
Twins (%)	12 (12%)^c^
Caesarean section (%)	43 (43%)^c^
Caucasian (%)	90 (91%)^c^

Data are shown as ^a^mean (SD), ^b^median (IQR) or ^c^counts (percentages), SD standard deviation, IQR interquartile range

Mean (SD) nostril size off all investigated infants was 5.3mm (0.6). There was a significant correlation between nostril size and birthweight (r=0.34, p<0.001), GA (r=0.27, p<0.05) and length at birth (r=0.32, p<0.001).

The ODs of ETTs differed widely depending on the manufacturer (Table 1), e.g. the OD of a 3.5mm ETT ranged from 4.8mm (Portex ®) to 5.3mm (Rüsch®). Some OD of a 3.5mm ETT were even smaller than the OD of a 3.0mm ETT (e.g. the 3.0mm ETT from Rüsch® has an OD of 5.0mm while the 3.5mm ETT from Portex® has an OD of 4.8mm).

**Fig. 2:**
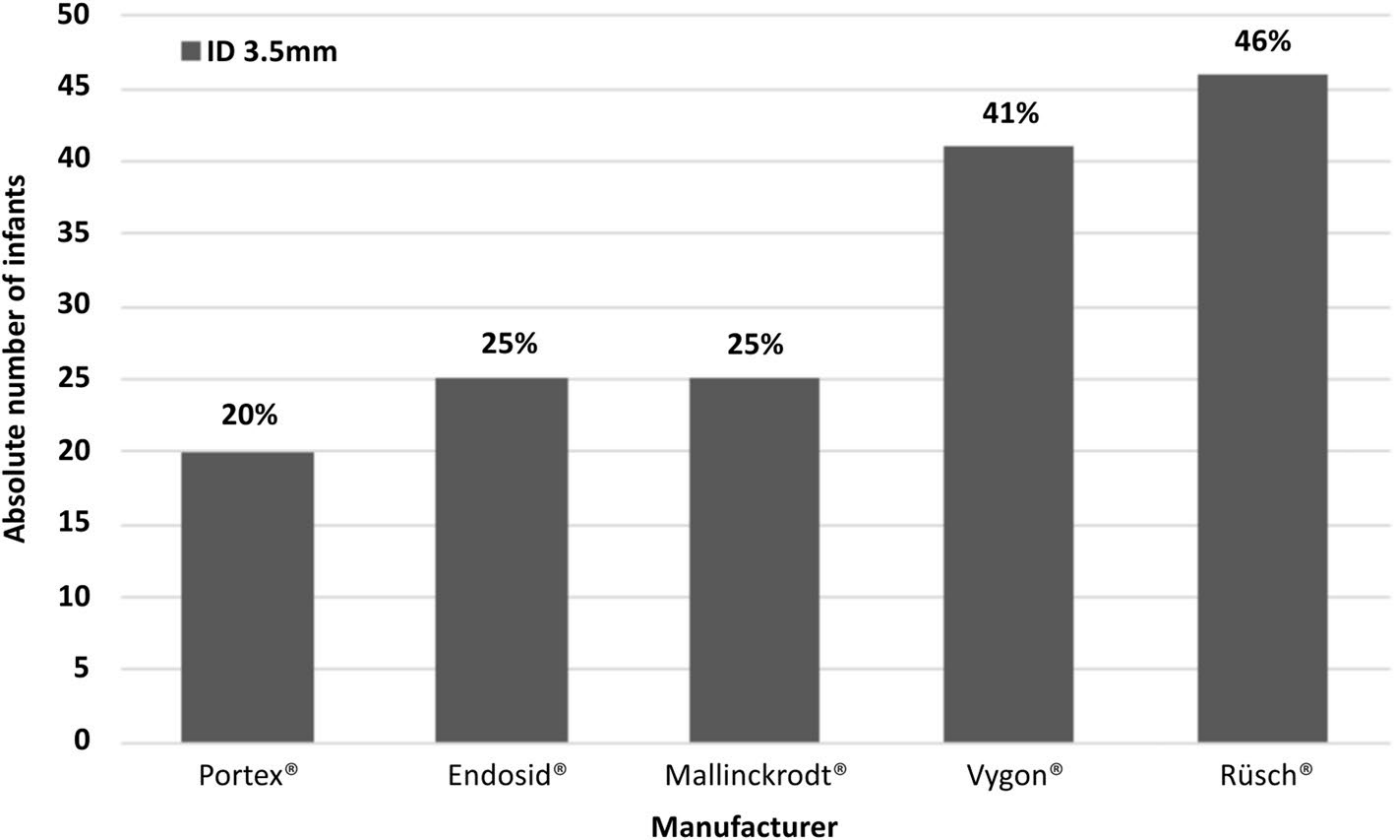
Absolute number of infants and percentage of infants in whom the OD of the ETT with an ID of 3.5 was bigger than the infant’s nostril size based on recent ERC guideline 2021

Figure 2 shows the percentage of infants in whom the OD of the ETT was larger than the infant’s nostril size depending on the manufacturer (based on the ERC guidelines from 2021 that recommend a 3.5mm ETT).

A 3.5mm ETT would be too large for 20-46% of the infants depending on the manufacturer.

Infants with a birthweight <3000g (n=45) had a mean (SD) nostril size of 5.1mm (0.7) and infants with a birthweight ≥3000g (n=54) a mean nostril size of 5.4mm (0.6).

**Fig. 3:**
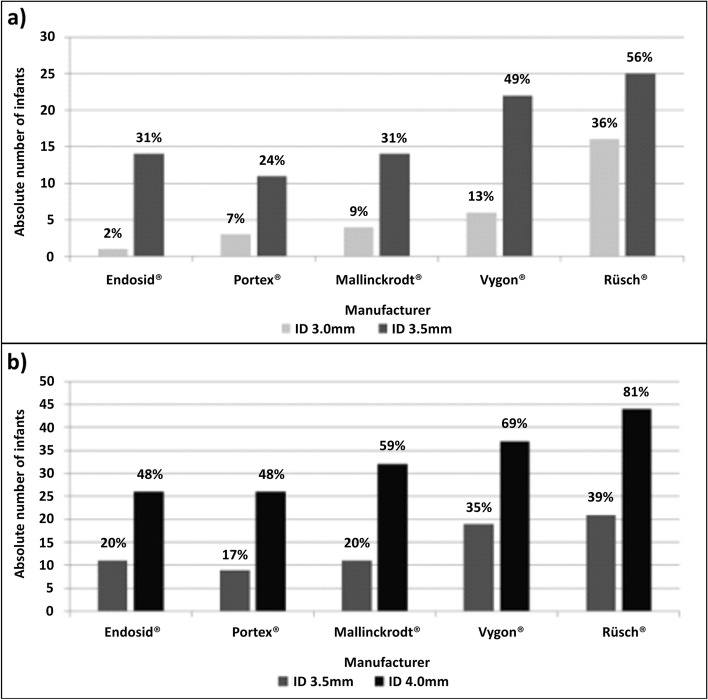
**a** Absolute number of infants and percentage of infants with a birthweight <3000g in whom the OD of the ETT with an ID of 3.0 and 3.5mm was bigger than the infant’s nostril size **b** Absolute number of infants and percentage of infants with a birthweight ≥3000g in whom the OD of the ETT with an ID of 3.5 and 4.0mm was bigger than the infant’s nostril size

Figure 3 show the percentage of infants divided into two weight groups (<3000g and ≥3000g respectively) in whom the OD of the ETT was larger than the infant’s nostril size depending on the manufacturer (based on the recommendation from the ILCOR statement from 1999).

For infants with a birthweight <3000g (Fig. 3a) a 3.0mm ETT would be too large for 2-36% of the infants, and a 3.5mm ETT for 24-56%.

For infants with a birthweight ≥3000g (Fig. 3b) a 3.5mm ETT would be too large for 17-39% of the infants, and a 4.0mm ETT would be too large for 48-81% of all measured infants, depending on the manufacturer.

## Discussion

We compared the OD of ETTs from different manufacturers with the nostril sizes of late preterm and term infants to evaluate which tube is suitable to pass the nostrils.

Recent guidelines only make recommendations on the appropriate tube size based on ID [7–11] Moreover, available recommendations are not based on evidence. Since nasal intubation is common in newborn infants and the first narrow part to pass the tube is the nostril, we measured nostril sizes of term and late preterm infants and compared them with the OD of ETTs of different manufacturers.

The OD differs widely depending on the manufacturer and therefore some ETTs seem easier to pass the nostrils than others. Depending in the manufacturer, the OD of the ETT was larger than the nostril in 20-46% when following the current ERC recommendation [7]. Therefore, clinicians should be aware of the widely varying ODs among manufactures and consider using a smaller ETT in infants <3000g, if the available ETT in their unit has a larger OD.

Up to 50% of intubation attempts by paediatric residents are unsuccessful upon the first approach [17] and up to 14% of neonatal intubations are considered difficult, requiring three or more attempts potentially leading to severe desaturations [18, 19].

The occurrence of a difficult intubation in neonates is hard to predict due to the lack of sensitivity of bedside screening tests [18]. Beside inexperience, factors contributing to a difficult intubation might be excessive secretions [20], syndromes with limited mouth opening [21], micrognathia, retrognathia and cleft palate [22] or limited neck extension. For the difficult intubation, technologies such as video laryngoscopy may help to implement better success rates [23].

There are no guidelines on the route of endotracheal intubation, either orally or nasally, and the relevant Cochrane review showed no differences in complication rates [1].

An analysis of intubations in paediatric intensive care units demonstrated that the nasal intubation route is rarely used in the United States, but nasal intubation is associated with a lower rate of unplanned extubations [24].

During nasal intubation the first constricting point is the posterior nasal aperture, connecting the nasal cavity with the pharyngeal cavity via the nasopharynx [25], which might delay the intubation procedure. During oral intubation, this first constricting point in the nasal cavity is omitted.

An additional constricting point equally concerning either intubation route is the inner diameter of the cricoid cartilage [4] as the posterior cricoid lamina has a "V" configuration [26]. Postintubation lesions might occur at two levels, the posterior part of the glottic plane with risk of vocal necrosis and the subglottic level with risk of subglottic stenosis [13]. Post-mortem studies demonstrated that in most preterm infants the cricoid ring is smaller than the tracheal diameter [5]. In theory, the cricoid cartilage is rigid, which impairs its adaptation to a ETT size larger than its diameter [26]. However, some laryngeal elasticity exists in preterm infants allowing intubation with larger tube sizes than the anatomical structure would predict. This elasticity disappears when approaching 40 weeks’ gestation [13]. This missing elasticity in term infants indicates that the OD of an ETT should not be too large in term infants.

Strengths of our study are the good balance in the number of participants weighing ≥/<3000g and an altogether high number (n=99) of investigated infants from 34 to 41 weeks’ gestation with a birthweight ranging from 1850g to 4100g, showing nostril sizes with small standard deviation.

Limitations include that only externally visible anatomical structures such as the nostril size were used for this analysis, and we can only speculate about the internal anatomical constricting areas such as the posterior naris or the cricoid ring.

Nevertheless, the results of this study show that recommendations of ETT size should not only consider the ID but also the OD. Future studies should investigate which ETT ODs should be used in late preterm und term infants with the aim to be small enough to pass the nostrils and the cricoid ring but without causing air leak.

Our data might support clinicians performing nasal intubation in near term and term infants with more information about the optimal ETT to pass the nostrils.

## Conclusion

An 3.5mm ETT might cause difficulties in passing the nasal cavity of late preterm and term infants in 20-41% depending on the manufacturer. Clinicians should be aware of the OD of ETTs to reduce unsuccessful nasal intubation attempts caused by ETT sizes not fitting the nasal cavity. Generated data comparing OD of ETT with nostril size may help to adapt recommendations in future.

## Data Availability

All data relevant to this subgroup analysis are included in the article.

## Abbreviations

CS: Choanal stenosis
ETT: Endotracheal tube
GA: Gestational age
ID: Inner diameter
SD: Standard deviation
OD: Outer diameters
